# Adults with autism spectrum disorders exhibit decreased sensitivity to reward parameters when making effort-based decisions

**DOI:** 10.1186/1866-1955-4-13

**Published:** 2012-05-21

**Authors:** Cara R Damiano, Joseph Aloi, Michael Treadway, James W Bodfish, Gabriel S Dichter

**Affiliations:** 1 Department of Psychology, University of North Carolina at Chapel Hill, CB# 3270, Davie Hall, UNC-CH, Chapel Hill, NC, 27599-3270, USA; 2 Department of Psychology, Vanderbilt University, PMB 407817 2301 Vanderbilt Place, Nashville, TN, 37240-7817, USA; 3 McLean Hospital/Harvard Medical School, 115 Mill Street, Belmont, MA, 02478, USA; 4 Department of Psychiatry, University of North Carolina at Chapel Hill School of Medicine, CB# 7160, Chapel Hill, NC, 27599-7160, USA; 5 Carolina Institute for Developmental Disabilities, University of North Carolina at Chapel Hill School of Medicine, CB# 7255, 101 Manning Drive, Chapel Hill, NC, 27599-7255, USA

**Keywords:** Reward, Motivation, Decision-making, Autism spectrum disorders, Dopamine

## Abstract

**Background:**

Efficient effort expenditure to obtain rewards is critical for optimal goal-directed behavior and learning. Clinical observation suggests that individuals with autism spectrum disorders (ASD) may show dysregulated reward-based effort expenditure, but no behavioral study to date has assessed effort-based decision-making in ASD.

**Methods:**

The current study compared a group of adults with ASD to a group of typically developing adults on the Effort Expenditure for Rewards Task (EEfRT), a behavioral measure of effort-based decision-making. In this task, participants were provided with the probability of receiving a monetary reward on a particular trial and asked to choose between either an “easy task” (less motoric effort) for a small, stable reward or a “hard task” (greater motoric effort) for a variable but consistently larger reward.

**Results:**

Participants with ASD chose the hard task more frequently than did the control group, yet were less influenced by differences in reward value and probability than the control group. Additionally, effort-based decision-making was related to repetitive behavior symptoms across both groups.

**Conclusions:**

These results suggest that individuals with ASD may be more willing to expend effort to obtain a monetary reward regardless of the reward contingencies. More broadly, results suggest that behavioral choices may be less influenced by information about reward contingencies in individuals with ASD. This atypical pattern of effort-based decision-making may be relevant for understanding the heightened reward motivation for circumscribed interests in ASD.

## Background

Clinical observations suggest that individuals with autism spectrum disorders (ASDs) may have reduced motivation to seek social interaction, yet heightened motivation to expend effort in the pursuit of certain non-social stimuli (that is, circumscribed interests). Consistent with these observations, some theoretical and clinical conceptualizations of ASD highlight a failure to assign reward value to social interactions originating in infancy (example, [1-3]). According to such models, early decreased social reward motivation may lead to deficits in basic social and language abilities [4].

The high prevalence of circumscribed interests in this population further suggests that ASD is characterized by not only diminished motivation for social stimuli but also atypical motivation for idiosyncratic non-social stimuli as well [5-7]. More generally, reward processing atypicalities for both social and non-social stimuli have been evidenced in ASD through behavioral, psychophysiological, and neuroimaging studies in this population [8-16].

Although several different approaches have been used to measure the functional output of reward processing systems in ASD, reward-based choices in the context of effort expenditure have yet to be examined in this population. Effort-based decision-making indexes the behavioral motivation to obtain rewards relative to effort expenditure and missed opportunities for competing rewards (that is, reward-based cost-benefit decisions). This process is critically important for choosing among biologically relevant goal-directed behaviors, such as feeding, learning, and social interactions [17-19]. Preclinical data indicate that effort-based decision-making is one sub-component of reward processing that is mediated in part by the mesolimbic dopamine system [20,21]. More specifically, animal studies suggest that dopaminergic activity in the ventral striatum (and more specifically the nucleus accumbens (NAc)) mediates how expected reward value and effort expenditure influence behavioral choices [22-24]. Depletion or reduction of dopaminergic activity in the NAc diminishes the tendency to expend effort to obtain a reward [25,26]. In other words, NAc dopamine appears to influence the tendency to choose behaviors that offer greater reward requiring greater effort expenditure [27]. In this regard, ventral striatal dopamine depletions appear to shift the cost-benefit gradient that governs effort-based decision-making such that an animal with intact dopaminergic functioning expends greater amounts of effort for larger rewards whereas animals with ventral striatal dopamine depletion choose lower-effort, smaller-reward options. Importantly, alterations in reward processing within ventral striatal regions have been reported in ASD [9,10,16].

Despite accumulating evidence suggesting reward processing atypicalities in ASD, no research to date has investigated decisions related to effort-based reward motivation in ASD. Previous research has demonstrated impaired decision-making in individuals with ASD [11,28,29], yet no study to date has examined decision-making in the context of varying degrees of reward magnitude, reward probability, and effort expenditure to obtain rewards. The goal of the present study was to investigate choices in the context of varying rewards and behavioral effort expenditure in ASD via an effort-based decision-making task, the Effort Expenditure for Rewards Task (EEfRT). The EEfRT requires participants to choose between an “easy task” or a “hard task” under varying reward probabilities to earn different amounts of monetary rewards. The EEfRT was originally developed to mimic seminal animal studies demonstrating the role of mesolimbic dopaminergic functioning in effort-based decision-making [30], and human studies supporting the sensitivity of this task to the functional output of mesolimbic dopamine systems. Specifically, there is evidence that EEfRT performance is related to individual differences in symptoms of anhedonia (that is, decreased sensitivity to rewards; [31]) and is modulated by administration by *d*-amphetamine, a dopamine agonist [32]. The EEfRT measures the capacity to make efficient decisions by dynamically responding to changing information about reward probability and magnitude.

Based upon empirical evidence of reward processing impairments in dopaminergic projection regions involved in effort-based decision-making [8-12,15,16] as well as evidence for impaired decision-making in ASD [11,28,29], we hypothesized that individuals with ASD would demonstrate atypical decision-making related to effort expenditure for rewards that would be characterized by decreased sensitivity to reward probabilities and effort contingencies, relative to neurotypical control participants. Because individuals with ASD may be relatively more willing to expend effort for some non-social stimuli [6,33,34], we further hypothesized that effort-based decision-making profiles would be related to circumscribed interests in ASD, as circumscribed interests have been linked to reward system functioning in previous studies [9,35].

## Methods

### Participants

Participants included 20 adults with ASD (male/female: 17/3; age: *M* = 25.95, *SD* = 7.96, range = 18 to 48) and 38 typically developing controls (male/female: 34/4; age: *M* = 20.42, *SD* = 5.64, range = 18 to 43). Exclusion criteria for both groups included known motor or sensory deficits and an intelligence quotient (IQ) score ≤ 85 (Wechsler Abbreviated Scales of Intelligence) [36]. Participants with ASD were recruited from the Autism Subject Registry maintained through the University of North Carolina (UNC) Carolina Institute for Developmental Disabilities. Twelve participants were diagnosed with high-functioning autism and eight participants were diagnosed with Asperger’s syndrome. All ASD diagnoses were made according to expert clinical judgment of licensed clinical psychologists experienced in ASD diagnoses and confirmed by standard algorithm cutoff scores for a diagnosis of Autism Spectrum Disorder on Module 4 of the Autism Diagnostic Observation Schedule-Generic (ADOS-G) [37]. Control participants were recruited via the Human Participation in Research Subject Pool maintained by the UNC Department of Psychology and from a database of control participants maintained by our laboratory. The Autism-Spectrum Queotient (AQ) [38] was used to screen controls for high levels of autism symptomatology, and all control participants scored below the recommended cutoff for ASD on the AQ (≤ 32; *M =* 14.05, *SD* = 6.19*,* range = 3 to 29).

Groups did not differ in Verbal IQ, Performance IQ, Full Scale IQ, handedness (as measured by the Edinburgh Handedness Inventory [39] or gender ratios, all *p*’s > .10 (see Table 1). However, ASD participants were significantly older than control participants (ASD: *M* = 25.95, *SD* = 7.96; Control: *M* = 20.42, *SD* = 5.64), Welch’s t(29.31) = 2.76, *p* = 0.01. When age was entered into analyses as a covariate, there were no significant main effects or interactions involving age on any dependent measures in all analyses that included diagnostic group as a factor (see the Additional file 1: Supplementary Materials). Thus, age was not included as a covariate in the analyses reported below.

**Table 1:** Means (and Standard Deviations) scores on demographic and clinical measures for the ASD and Control Groups

	**ASD (n = 20)**	**Control (n = 38)**	***t / χ*^*2*^**	***p***
	**Mean (SD)**	**Mean (SD)**		
Age	25.95 (7.96)	20.42 (5.64)	2.764	0.01
Verbal IQ	112.50 (14.65)	112.94 (9.94)	-0.135	0.89
Performance IQ	113.60 (10.39)	109.17 (9.06)	1.664	0.10
Full Scale IQ	114.70 (11.25)	112.82 (8.66)	0.709	0.48
Handedness (Absolute Value) ^a^	68.55 (24.48)	74.72 (21.00)	-1.004	0.32
Right: Left: Ambidextrous	16:1:3	31:2:5	0.038	0.98
Male: Female ratio	17:3	34:4	0.247	0.62
IRB Insistence on Sameness	5.61 (3.85)	1.74 (1.70)	4.082	<0.01
Range	0-14	0-5		
IRB Circumscribed Interests	8.17 (2.88)	2.18 (2.05)	8.927	<0.01
Range	4 to 13	0 to 9		
IRB Motor Stereotypies	6.06 (3.64)	2.66 (2.39)	3.612	<0.01
Range	0 to 11	0 to 8		
ADOS SBRI	1.68 (1.62)	N/A	N/A	N/A
Range	0 to 5			
ADOS Communication	3.95 (1.36)	N/A	N/A	N/A
Range	2 to 7			
ADOS Social Interaction	8.35(2.70)	N/A	N/A	N/A
Range	4 to 14			

^a^Handedness was indexed with the Edinburgh Handedness Inventory [39]. For each participant, this questionnaire provided handedness as both a continuous value (from −40 to 40, with larger negative values indicating a stronger preference for the left hand and larger positive values indicating a stronger right hand preference) and a categorical value (left-handed: <−40, ambidextrous: ≤-40 and ≤+40, and right-handed: > + 40). The absolute value of handedness is an index of the degree of ambidexterity regardless of right or left hand preference (with values closer to 0 indicating greater ambidexterity). The ADOS was only completed with the ASD group. ADOS, Autism Diagnostic Interview.

IRB = Interview for Repetitive Behaviors.

SBRI, Stereotyped Behaviors and Restricted Interests.

### Measures

#### Effort-expenditure for rewards task (‘EEfRT’)

**Figure 1:**
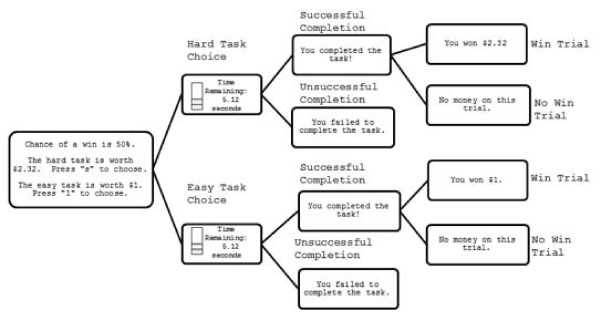
**Schematic diagram of the Effort Expenditure for Rewards Task (‘EEfRT’).** A) Participants were presented with information regarding the reward magnitude of the hard task for that trial, and the probability of receiving any reward for both the easy and hard tasks. B) Participants chose the easy or hard task by pressing a designated key. C) Participants made rapid button presses to complete the chosen task for seven seconds (easy task) or 21 seconds (hard task). D) Participants received feedback on whether they have completed the task. E) Participants received reward feedback indicating whether they received any money for that trial.

The EEfRT measures the willingness to expend effort to obtain a monetary reward under different conditions of reward probability and reward magnitude [31]. All participants completed 50 trials. In each trial, participants were asked to choose between an ‘easy task’ and a ‘hard task’ (Figure 1). It was emphasized that successful trial completion did not guarantee winning money (a ‘win trial’) but rather it was possible that successful completion could result in not wining money (a ‘no win’ trial). Before making this choice, participants were provided with two pieces of information that varied from trial to trial: 1) *reward probability* (12%, 50%, or 88%) of a ‘win’ or ‘no win’ trial upon successful trial completion, and 2) *reward magnitude* for successfully completed ‘win’ trials. Reward magnitudes were $1.00 for successful completion of easy task ‘win’ trials and one of 17 levels of reward magnitude ranging from $1.24 to $4.12 for successful hard task ‘win’ trials. Trials were presented in the same randomized order for every participant.

Successful completion of the easy task required 30 repeated button presses in seven seconds on a standard keyboard using the dominant index finger, while successful completion of the hard task required 100 presses with the non-dominant ‘pinky’ finger in 21 seconds. With each press a line was added to a rectangle onscreen and participants were informed that they would be eligible to win money for each trial if they raised this bar to the top of the rectangle. The time remaining was also displayed throughout the trial. Following the task, participants were informed whether they completed the task successfully and, if they had completed it successfully, how much money they won. They were also informed that the summed value of two randomly-selected win trials would be given as a bonus ($2.00 to $8.24) in addition to the base rate compensation ($10). The EEfRT was modified so that participants had an unlimited amount of time to make choices to accommodate potential slower processing speeds in the ASD group [40,41].

#### Autism Diagnostic Observation Schedule (ADOS)

The ADOS is a standardized assessment for observing behavior relevant to autism symptomatology. The standard algorithm cutoff scores from Module 4 of this measure were used to inform diagnostic evaluations or confirmations.

#### Interview for Repetitive Behavior (IRB)

The IRB [42] is a semi-structured clinical interview designed to measure the occurrence of a variety of types of repetitive behaviors and interests and their severity in individuals with ASD. Trained interviewers rated the frequency, intensity, interference, and accommodation needed for each of these behaviors and interests based upon participants’ descriptions. Scores were derived for motor stereotypies, insistence on sameness, and circumscribed interests with higher scores indicating greater severity and functional impairment. These scores were then used in hierarchical regression analyses examining the relationship between EEfRT performance and repetitive behavior measures.

### Data analysis strategy

Primary analyses investigated whether the ASD group differed from the control group in the willingness to expend effort to obtain uncertain rewards via a 3 (Reward Magnitude: small, medium, large) x 3 (Probability: 12%, 50%, 88%) x 2 (Group: ASD, control) repeated measures analysis of variance (ANOVA). Levels of the Reward Magnitude factor were grouped in the following manner: small was defined as any value between $1.24 and $2.00, medium as values between $2.01 and $3.00, and large as values between $3.00 and $4.12. The dependent variable (that is, the willingness to expend effort for rewards) was the percentage of times the hard task was chosen across all trials of the EEfRT task (including all levels of Probability and Reward Magnitude). These analyses were supplemented by a 2 (Reward Magnitude: small, large) x 2 (Probability: 12%, 88%) x 2 (Group: Autism, Control) ANOVA with lower degrees of freedom. These analyses simply excluded data from the medium Reward Magnitude condition (which as in the analyses described above, was any value between $2.01 and $3.00) and from the 50% Probability condition, in order to examine the pattern of results when only the more extreme Reward Magnitude and Probability levels were included. Between-group *t*-tests were also conducted to examine potential group differences in variables related to task performance but unrelated to the effort-based decision-making construct (that is, the average response time in choosing between tasks and the number of successfully completed trials).

Secondary analyses examined the extent to which individuals with ASD differed from controls in response flexibility since several studies have found cognitive flexibility impairments in ASD [43-45]. A 2 (Preceding Trial Outcome: win, loss) × 2 (Group: ASD, control) repeated measures ANOVA was conducted with the dependent variable of the percentage of times that each participant changed his or her response on a given trial from the response given on the preceding trial.

Finally, relations between EEfRT performance and repetitive behaviors symptoms (motor stereotypies, insistence on sameness, and circumscribed interests) were evaluated by examining the relationship between the percentage of hard task choices and scores from the IRB. Hierarchical multiple regression analyses were conducted in which Group was entered first followed by repetitive behavior measures.

## Results

### Percentage of hard task choices

**Figure 2:**
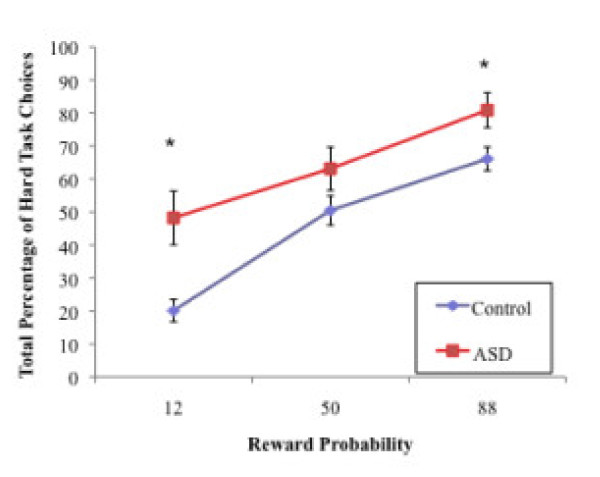
**The mean percentage of hard task choices by reward probability for the ASD and Control groups.** Significant group differences were detected at the 12% probability level, *t*(57) = 3.31, *p* = 0.003, and the 88% probability level, *t*(57) = 2.37, *p* = 0.02. The error bars indicate +/− 1 SEM. ASD, Autism Spectrum Disorder; SEM, standard error of the mean.

**Figure 3:**
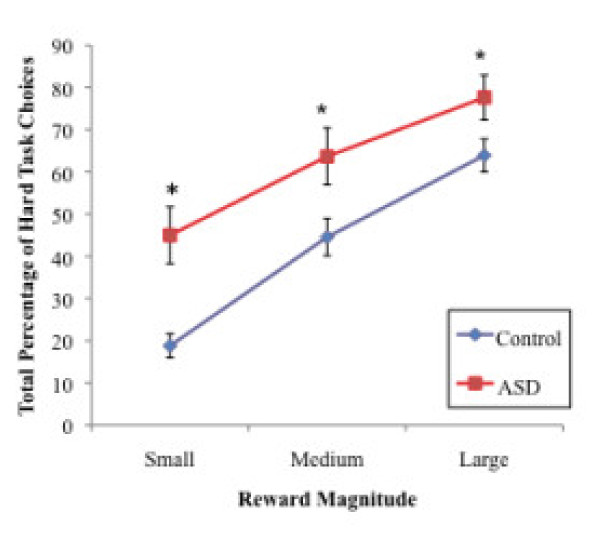
**The mean percentage of hard task choices by reward magnitude for the ASD and Control groups.** Significant group differences were detected for small rewards, *t*(57) = 3.60, *p* = 0.001, medium rewards, *t*(57) = 2.45, *p* = 0.02, and large rewards, *t*(57) = 2.07, *p* = .04. The error bars indicate +/− 1 SEM. ASD, Autism Spectrum Disorder; SEM, standard error of the mean.

The omnibus 3 (Reward Magnitude: small, medium, large) × 3 (Probability: 12%, 50%, 88%) x 2 (Group: ASD, control) repeated measures ANOVA performed on the percentage of hard task choices revealed a significant three-way interaction, *F*(4, 53) = 4.12, *p* = 0.006, and a significant Probability x Group interaction, *F*(2, 55) = 44.81, *p* = 0.003. However, the Reward Magnitude x Group interaction was not significant, *F*(2, 55) = 1.73, *p* = .19. Significant main effects were detected for Probability, *F*(2, 55) = 44.81, *p* < 0.001, Reward Magnitude, *F*(2, 55) = 69.16, *p* < 0.001, and Group, *F*(1, 56) = 10.64, *p* = 0.002. The main effect of Group reflects a greater percentage of hard task choices overall in the ASD (*M =* 64.08, *SD* = 24.53) versus the control (*M =* 45.57, *SD* = 20.39) group. This finding suggests that individuals with ASD demonstrated a greater willingness to expend effort for rewards across all trial types compared to controls. Between-groups *t-*tests also revealed a significantly greater proportion of hard task choices in the ASD group versus the control group at each probability levels and all reward value levels, all *p*’s < 0.05, except at the 50% level, *t*(56) = 1.66, *p* = 0.10 (see Figures 2 and 3). A 2 (Reward Magnitude: small, large) × 2 (Probability: 12%, 88%) × 2 (Group: Autism, Control) ANOVA (in which data from the medium Reward magnitude and 50% Probability conditions were excluded from analyses) yielded the same pattern of results.

Next, 3 (Reward Magnitude: small, medium, large) × 2 (Group: ASD, control) repeated measures ANOVAs were conducted separately for each level of probability on the percentage of hard task choices. For 12% probability trials, this interaction effect was not significant, *F*(2, 55) = 1.32, *p* = 0.27. Yet, main effects of Reward Magnitude, *F*(2, 55) = 19.89, *p* < 0.001, and of Group were detected, *F*(2, 55) = 15.41 *p* < 0.001. For the 50% probability trials, the interaction was not significant, *F*(2, 55) = 1.53, *p* = 0.23, and neither was the main effect of Group, *F*(2, 55) = 3.90, *p* = 0.05, yet there was a significant main effect of Reward Magnitude, *F*(2, 55) = 44.30, *p* < 0.001. For 88% probability trials, there were a significant Reward Magnitude x Group interaction, *F*(2, 55) = 8.08, *p* = 0.001, and significant main effects of Reward Magnitude, *F*(2, 55) = 51.12, *p* < 0.001, and of Group, *F*(2, 55) = 6.32, *p* = 0.015. These results indicate that reward magnitude significantly influenced effort-based decision-making at all levels of probability across both groups while group differences in decision-making only emerged at the 12% and 88% probability levels. In addition, at the 88% probability level, reward magnitude had a different influence in the ASD versus control groups: in this condition the ASD group was more likely to choose the hard task for a small reward, *t*(56) = −4.03, *p* < 0.001 (ASD: *M* = 67.00, *SD* = 33.26; Control: *M* = 32.63, *SD* = 29.56), while no group differences were found for medium or large rewards. In other words, responses of individuals in the control group were moderated by both magnitude and probability (demonstrated by this group’s tendency to choose the easy task more often when reward magnitude was small even if probability was large), while individuals with ASD chose the hard task even in the context of a small reward.

Next, 3 (Reward Magnitude: small, medium, and large) × 3 (Probability: 12%, 50%, 88%) repeated measures ANOVAs on percentage of hard task choices within each group revealed significant main effects of Probability, *F*(2, 36) = 74.97, *p* < 0.001, and Reward Magnitude, *F*(2, 36) = 66.50, *p* < 0.001, in the control group, as well as main effects of Probability, *F*(2, 18) = 9.02, *p* = 0.002, and of Reward Magnitude, *F* (2, 18) = 21.69, *p* < 0.001, in the ASD group. The main effects of Probability and Reward Magnitude in both groups suggest that the percentage of hard task choices was modulated by probability and reward level in both the ASD and control groups. However, the significant Reward Magnitude × Probability × Group interaction reported earlier indicates that diagnostic group moderated the degree of these effects on effort-based decision-making, suggesting that the control group was more influenced by reward parameters, such as magnitude and probability, when compared to the ASD group.

### Response latency and EEfRT success

There were no group differences in average response times for choosing between the easy and hard tasks, Welch’s *t*(19.76) = 1.60, *p* = 0.13 (ASD: *M* = 5.96 s, *SD* = 7.52; Control: *M* = 3.24 seconds, *SD* = 1.46), or in the percentage of trials completed successfully, Welch’s *t*(20.89) = 1.92, *p* = 0.07 (ASD: *M* = 89.30%, *SD* = 18.82; Control: *M* = 97.58%, *SD* = 5.75). In addition, the primary dependent variable (percentage of hard task choices) was not correlated with either the average response times, *r*(58) = 0.60, *p* = 0.65, or the percentage of trials completed successfully, *r*(58) = −.15, *p* = 0.26.

### Response flexibility

To analyze response flexibility during effort-based decision-making, a 2 (Preceding Trial Outcome: win, loss) x 2 (Group: ASD, control) repeated measures ANOVA was conducted to examine the dependent variable of the percentage of trials in which participants changed their response from the preceding trial. This analysis revealed no significant main effects of Group, *F*(2, 56) = 2.51, *p* = 0.12, or Preceding Trial Outcome, *F*(2, 56) = 3.06, *p* = 0.09, and no significant interaction, *F*(2, 56) = .065, *p* = 0.43 (see Table 2). These analyses demonstrate that individuals with ASD did not differ significantly in response flexibility from their typically developing counterparts (for example, they were not simply ‘stuck’ on one task choice) and that the ASD group was not significantly more influenced by previous trial outcome than the control group.

**Table 2:** Means (and Standard Deviations) of the Percentage of Trials in which the Response Changes from the Preceding Trial for All Trials Overall, Trials Following a Loss, and Trials Following a Win in the ASD and Control Groups

	**ASD**	**Control**	***t***	***p***
	**Mean (SD)**	**Mean (SD)**		
**Overall Percent of Trials with Response Change**	35.80 (17.55)	42.11 (9.97)	1.49	0.15
**Percent of Trials with Response Change After Loss**	37.63 (16.85)	42.70 (12.06)	1.33	0.19
**Percent of Trials with Response Change After Win**	41.46 (11.18)	33.82 (20.21)	1.57	0.13

ASD, Autism Spectrum Disorder.

### The relationship between EEfRT performance and repetitive behaviors

**Figure 4:**
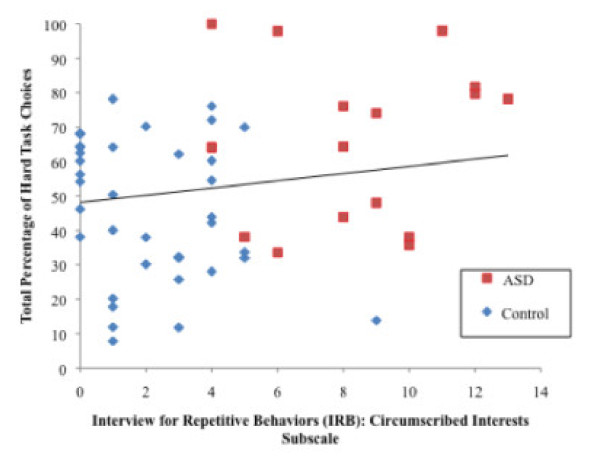
**Scatterplot of the relation between the Circumscribed Interests subscale of the Interview for Repetitive Behavior (IRB) and the mean percentage of EEfRT hard task choices.** The error bars indicate +/− 1 SEM. EEfRT, Effort Expenditure for rewards Task; SEM, standard error of the mean.

Separate hierarchical multiple regression analyses were conducted to examine the extent to which symptoms of repetitive behaviors and restricted interests from the IRB were related to the dependent variable of total percentage of hard task choices. Each of three separate models included Group entered as the first predictor, then one of the three repetitive behavior severity subscales from the IRB (Motor Stereotypies, Insistence on Sameness, and Circumscribed Interests) entered as a secondary predictor and the percentage of hard task choices as the dependent variable. As summarized in Table 3, the models containing the subscales of Motor Stereotypies and Insistence on Sameness did not account for a significant amount of variance after controlling for the variance explained by Group, with Motor Stereotypies accounting for only an additional 0.80% of the variance and Insistence on Sameness only accounting for 0.10%. However, in the third regression analysis, the addition of the Circumscribed Interest predictor to the model was significant, accounting for 7.00% of the variance in the dependent variable (see Figure 4). These results suggest that the tendency to have circumscribed interests is significantly related to an increased willingness to expend effort for rewards above and beyond the effect of diagnostic group.

**Table 3:** Summary from the Hierarchical Regression Analyses Examining the Amount of Variance in EEfRT Task Performance (the Percentage of Hard Task Choices) Accounted for by the Repetitive Behavior Variables from the IRB After Controlling for the Amount of Variance in EEfRT Task Performance Accounted for by Diagnostic Group

	**R**^**2**^	***▵F***	***p***
**Motor Stereotypies**	0.008	0.53	0.47
**Insistence on Sameness**	0.001	0.76	0.74
**Circumscribed Interests**	0.070	5.02	0.029

EEfRT, Effort Expenditure for Rewards Task; IRB, Interview for Repetitive Behavior.

## Discussion and conclusion

In the present study, we found that adults with ASD were relatively more likely to choose to expend increased effort for rewards and were more likely to demonstrate decreased sensitivity to reward probability and magnitude when making effort-based decisions. These group differences were not attributable to other factors that may have influenced task performance, such as task success or response flexibility. The EEfRT was adapted from a task designed to investigate neurobiological mechanisms of reward motivation in animals (that is, phasic dopamine release in the ventral striatum) [30], and has been linked to individual differences in symptoms of anhedonia [31] as well as individual differences in sensitivity to pharmacologic dopamine challenge [32]. Thus, these results suggest that individuals with ASD may have differential cost-benefit gradients in the context of monetary rewards with varying probabilities and magnitudes and that this atypical effort-based decision-making pattern is potentially linked to the functional output of dopaminergic systems. This is in contrast to reports of diminished motivation for social rewards in ASD [8,10,16], as our findings suggest that individuals with ASD demonstrate atypical patterns of reward-related performance in a context that is not specifically associated with social information processing. Thus reward-related deficits in ASD may be more general than earlier accounts of specific deficits in social motivation have suggested.

The significant Group x Probability x Reward Magnitude interaction effect on hard task choices suggests that the ASD group was not simply characterized by a greater willingness to expend effort for monetary rewards, but rather was also differentially influenced by reward magnitude and probability information in these effort-based decisions. This is noteworthy given the extant literature on impaired decision-making in ASD that has found that individuals with ASD may fail to make decisions that use task information to maximize rewards [11] and that individuals with ASD are less likely to consider the context in making choices about rewards or losses when compared to a control group [28]. Similarly, in a study using a large-scale search task in a ‘foraging room’, individuals with ASD were found to choose a less efficient searching strategy relative to controls, disregarding information about rewards and not using strategies that would minimize energy expenditure [46]. The present study extends this line of research to suggest that ASD is characterized by impaired use of reward magnitude and probability information when making reward-based decisions. Our finding that adults with ASD demonstrate inefficient effort-based decision-making characterized by decreased sensitivity to reward parameters is consistent with previous studies that have demonstrated reward processing deficits in ASD [8-16]. Our findings advance this growing body of literature by suggesting that a previously unexplored aspect of reward processing, effort-based decision-making, may be atypical in ASD as well. Whereas previous conceptualizations of autism have focused primarily on domain-specific processing of social information findings from this growing body of research on reward processing and reward-related neural circuitry in ASD also indicate impaired processing of non-social rewards in ASD.

In line with these findings, clinical observations of individuals with ASD also suggest atypical processing of non-social rewards in this population, as individuals with ASD often have circumscribed interests which appear to involve heightened motivation to engage in nonsocial activities in a persistent or repetitive manner [5,7,47]. In particular, individuals with ASD seem willing to expend effort to obtain their circumscribed interests regardless of the context of this activity. Analyses examining the relationship between performance on the EEfRT task and the repetitive behavior symptom domain of ASD were examined. These analyses revealed that a tendency to choose higher-effort tasks is associated with a greater severity of circumscribed interest symptoms as reported on the Interview for Repetitive Behavior. This highlights the potential relevance of effort-based decision-making to circumscribed interests.

In line with these findings, previous neuroimaging studies have found activation in reward processing circuitry during the presentation of stimuli that are common circumscribed interests in ASD [9,35]. The link between circumscribed interests and reward processing in ASD is further supported by clinical observations of the rewarding nature of circumscribed interests and the successful use of circumscribed interests as rewards in empirically supported interventions for ASD [33,34,48]. Interestingly, motivational processes are also an established part of repetitive behavior symptomatology in obsessive-compulsive spectrum disorders where atypical repetitive behaviors are maintained by heightened negative valence systems such as avoidance of anxiety [49]. In contrast, circumscribed interests as observed in ASD appear to be related to approach motivation. Further, our finding in this study that individuals with ASD are more willing to expend effort to obtain a reward may signal a bias towards persistent approach motivated behavior in ASD in general and not just in regard to decisions around circumscribed interests.

A limitation of this work is the inclusion of only high-functioning individuals in the ASD group. Future research will be needed to determine whether these results generalize to low-functioning individuals on the autism spectrum. Additionally, because participants in this study were adults, future research will be needed to address the developmental profiles of effort-based decision making in ASD. When considered in a developmental context, it is possible that atypical effort expenditure for non-social rewards may be linked in some way to the early deficits in social motivation observed in ASD. However, because of the cross-sectional nature of this study, it is not possible to evaluate potential causal relations between aberrant effort-based decision-making and reduced social motivation in ASD. Another limitation of this study is the use of monetary rewards, given that monetary transactions may imply a social exchange and that social status is often ascribed to monetary gain. However, it is important to note that monetary rewards have a significant effect on behavioral choices and activate overlapping brain regions as primary rewards [50,51]. Future research examining choices to obtain rewards related to social interaction and to circumscribed interests will be important for understanding the role of effort-based decision-making in ASD.

In the absence of direct measures of brain function, it is possible that group differences in task performance were attributable to other factors unrelated to reward processing atypicalities. In this regard, however, it is important to consider previous findings of an association between EEfRT performance and a diminished response to rewards [31], as well as the influence of a dopamine agonist on EEfRT performance [32]. The lack of group differences in response flexibility, response latency, or the number of successful trials suggest that group differences were due to reward contingencies, rather than differential responding strategies or task engagement in the ASD group.

The increased tendency for higher-effort behavioral choices in ASD may have implications for behavioral interventions in this population. Some of the most effective interventions for ASD have been designed to motivate individuals with ASD to expend greater effort by manipulating the consistency or saliency of rewards [52-54]. Other successful programs have leveraged circumscribed interests to alter behavior [33,34,48]. Because children with ASD may respond less to social rewards (such as, teacher attention) or non-social rewards that are not salient to them (such as, grades), reward-based interventions for children with ASD often facilitate reward understanding in the context of learning. Yet, even with this type of support, there is significant heterogeneity in ASD with respect to response to reward-based interventions [55-58] and reward sensitivity may be an important predictor of success in such interventions [57]. Future research on this topic could investigate the relationship between intervention success and measures of reward motivation, such as effort-based decision-making.

In summary, the findings presented here provide evidence for altered reward-based decision-making in ASD. Task performance in the ASD group was characterized by a tendency to choose higher-effort tasks even in the context of lower reward probabilities and magnitudes. This inefficient strategy may hinder the ability of individuals with ASD to optimally engage or disengage with their environment and may ultimately contribute to the emergence or maintenance of symptoms associated with ASD. These results add to the growing body of literature implicating reward processing deficits in ASD and highlight the potential role of the mesolimbic dopaminergic system specifically and affective processing more generally in understanding ASD symptomatology.

## Abbreviations

ADOS-G: Autism diagnostic observation schedule – Generic; ANOVA: analysis of variance; ASD: Autism spectrum disorders; AQ: Autism-spectrum Quotient; EEfRT: Effort expenditure for rewards task; IRB: Interview for repetitive behaviors; IQ: Intelligence quotient; NAc: Nucleus accumbens; SD: Standard deviation; SEM: Standard error of the mean; SBRI: Stereotyped behaviors and restricted interests; UNC: University of North Carolina.

## Competing interests

The authors declare that they have no competing interests.

## Authors' contributions

JWB, MT, and GSD conceived of the study; CRD and JA collected and analyzed the data; CRD wrote the first draft of the manuscript; all authors read and approved the final manuscript.

## Supplementary Material

Additional file 1 Supplemental materials Click here for file
