# Supplementary material for: Adults with autism spectrum disorders exhibit decreased sensitivity to reward parameters when making effort-based decisions
Source: J Neurodev Disord. 2012 May 21;4(1):13. doi: 10.1186/1866-1955-4-13 (PMC3443445; doi:10.1186/1866-1955-4-13)
Supplement: Additional file 1 — Supplemental materials [file 1866-1955-4-13-S1.docx]

**Supplementary Materials**

Article Title: More EEfRT Than It’s Worth? Effort-Based Decision-making in Autism Spectrum Disorders

Journal: Journal of Neurodevelopmental Disorders

Authors: Cara Damiano ^A^, Joseph Aloi^A^ , Micheal Treadway ^B,C^,

James W. Bodfish ^D,E^, Gabriel S. Dichter ^A,D,E^

1. Department of Psychology, University of North Carolina at Chapel Hill, CB#3270, Davie Hall, Chapel Hill, NC 27599-3270
2. Department of Psychology, Vanderbilt University, PMB 407817 · 2301 Vanderbilt Place · Nashville, TN 37240-7817
3. McLean Hospital/Harvard Medical School, 115 Mill Street*,* Belmont, MA 02478
4. Department of Psychiatry, University of North Carolina at Chapel Hill School of Medicine, CB# 7160, Chapel Hill, NC 27599-7160.
5. Carolina Institute for Developmental Disabilities, University of North Carolina at Chapel Hill School of Medicine, CB# 3366, 101 Manning Drive, Chapel Hill, NC 27599-7160.

Corresponding author’s email: [cdamiano@email.unc.edu](mailto:cdamiano@email.unc.edu)

**Results including age as a covariate**

*Percentage of Hard Task Choices*

An omnibus 3 (Reward Magnitude: small, medium, large) x 3 (Probability: 12%, 50%, 88%) x 2 (Group: ASD, control) repeated measures ANOVA with age as a covariate was conducted on the proportion of hard task choices. This test revealed a significant interaction of Probability x Group, *F*(2, 54)=4.42, *p*=.02, and a significant Probability x Reward Magnitude interaction, *F*(2, 52)=2.98, *p*=.03. However, the Reward Magnitude x Group interaction was not significant, *F*(2, 54)=0.99 *p*=.38. Significant main effects were detected for Probability, *F*(2, 54)=6.86, *p*= .002, Reward Magnitude, *F*(2, 54)=8.73, *p*= .001, and Group, *F*(1, 55)= 10.81, *p*= .002.

For each probability level, 3 (Reward Magnitude: small, medium, large) x 2 (Group: ASD, control) repeated measures ANOVAs were performed with the dependent variable of percentage of hard task choices. For 12% probability trials, a main effect of Group was detected, *F*(1, 55)= 12.94,  *p*= .001. For the 50% probability trials, there was a main effect of Group, *F*(1, 55)=5.05, *p*=.03, and a main effect of Reward Magnitude, *F*(2, 54)=6.95, *p*=.002. For 88% probability trials, there was a significant Reward Magnitude x Group interaction, *F*(2, 54)=5.04, *p*=.01, and significant main effects of Reward Magnitude, *F*(2, 54)=9.81, *p*<.001, and of Group, *F*(1, 55)=6.93, *p*=.01.

*Response Flexibility*

A 2 (Preceding Trial Outcome: win, loss) x 2 (Group: ASD, control) repeated measures ANOVA with age as a covariate was conducted to examine the percentage of trials in which participants changed their response from the preceding trial. This analysis did not detect a significant interaction, *F*(1, 55)=0.28, *p*=.60, and or significant main effects of Group, *F*(1, 55)=0.06, *p*=.19, or Preceding Trial Outcome, *F*(1, 55)=0.007, *p*=.93.

*Correlations*

Partial correlations covarying for age found that the proportion of total hard task choices across both groups was negatively correlated with the number of circumscribed interests reported in the Interest Scale, *r*(45)= -.36, *p*= .013, and positively correlated with Insistence on Sameness subscale from the IRB, *r*(45)= .29 *p*= .047.
